# Open-source electronic data capture system offered increased accuracy and cost-effectiveness compared with paper methods in Africa

**DOI:** 10.1016/j.jclinepi.2014.06.012

**Published:** 2014-12

**Authors:** David G. Dillon, Fraser Pirie, Stephen Rice, Cristina Pomilla, Manjinder S. Sandhu, Ayesha A. Motala, Elizabeth H. Young, African Partnership for Chronic Disease Research (APCDR)

**Affiliations:** a International Health Research Group, Department of Public Health and Primary Care, University of Cambridge, Strangeways Research Laboratory, Wort's Causeway, Cambridge, CB1 8RN, United Kingdom; b Genetic Epidemiology Group, Wellcome Trust Sanger Institute, Wellcome Trust Genome Campus, Hinxton, Cambridge, CB10 1HH, United Kingdom; c Department of Diabetes and Endocrinology, Nelson R. Mandela School of Medicine, University of KwaZulu-Natal, Private Bag 7, Congella, 4013, Durban, South Africa; d System Support Team, Wellcome Trust Sanger Institute, Wellcome Trust Genome Campus, Hinxton, Cambridge, CB10 1HH, United Kingdom

**Keywords:** Sub-Saharan Africa, Data capture, Electronic questionnaire, Open-source, Epidemiology, Survey

## Abstract

**Objectives:**

Existing electronic data capture options are often financially unfeasible in resource-poor settings or difficult to support technically in the field. To help facilitate large-scale multicenter studies in sub-Saharan Africa, the African Partnership for Chronic Disease Research (APCDR) has developed an open-source electronic questionnaire (EQ).

**Study Design and Setting:**

To assess its relative validity, we compared the EQ against traditional pen-and-paper methods using 200 randomized interviews conducted in an ongoing type 2 diabetes case–control study in South Africa.

**Results:**

During its 3-month validation, the EQ had a lower frequency of errors (EQ, 0.17 errors per 100 questions; paper, 0.73 errors per 100 questions; *P*-value ≤0.001), and a lower monetary cost per correctly entered question, compared with the pen-and-paper method. We found no marked difference in the average duration of the interview between methods (EQ, 5.4 minutes; paper, 5.6 minutes).

**Conclusion:**

This validation study suggests that the EQ may offer increased accuracy, similar interview duration, and increased cost-effectiveness compared with paper-based data collection methods. The APCDR EQ software is freely available (https://github.com/apcdr/questionnaire).

## 1 Introduction

**What is new?**

• Existing electronic data capture options are often financially unfeasible in resource-poor settings or difficult to support technically in the field.
• To help facilitate large-scale multicenter studies in sub-Saharan Africa, the African Partnership for Chronic Disease Research (APCDR) has developed an open-source electronic questionnaire (EQ). This validation study compares the APCDR EQ with traditional pen-and-paper methods to assess the relative efficiency and accuracy of the EQ.
• Our results suggest that the EQ may offer increased accuracy, similar interview duration, and increased cost-effectiveness compared with paper-based data collection methods.
• The EQ software is freely available (https://github.com/apcdr/questionnaire).

In the past decade, electronic data capture systems have been increasingly adopted over traditional pen-and-paper methods for field survey data collection [1]. By combining the act of data collection with that of data input, electronic data capture devices may lower the cost and time of study monitoring and data management, as well as reduce the amount of data cleaning necessary on return from the field [2], [3], [4]. However, when doing research in a resource-poor setting such as sub-Saharan Africa (SSA), the use of electronic data capture systems may be problematic. Access to high-quality electronic devices and software may be financially unfeasible or restricted because of proprietary licensing, or existing software may be difficult to use for the creation of study-specific data collection forms and require specialist knowledge to maintain once in the field [2].

To address these limitations, the African Partnership for Chronic Disease Research (APCDR) has developed novel software to electronically capture questionnaire data in the field. This open-source data collection system was specifically designed to address the difficulties of working in SSA, and we anticipate that the electronic questionnaire (EQ) may provide an efficient and cost-effective alternative to traditional data collection methods. The validation study presented here aims to compare the APCDR EQ with traditional pen-and-paper methods to assess the relative efficiency and accuracy of the EQ.

## 2 Materials and methods

### 2.1 Study overview

We performed the EQ validation study between December 2012 and February 2013 within an ongoing type 2 diabetes case–control (T2DCC) study in Durban, South Africa. The default data collection method in the T2DCC study consisted of paper-based questionnaires and manual double entry of data into a single electronic database.

We validated the EQ in the case collection arm of the T2DCC study. Two hundred consecutive diabetes cases, eligible for inclusion in the T2DCC study, were randomized to be interviewed using either the EQ or the existing pen-and-paper methods. All 200 interviews were conducted by the same research nurse. After the EQ validation study was finished, the T2DCC study continued to recruit using existing pen-and-paper methods.

The EQ validation study was approved as a minor amendment to the T2DCC by the Biomedical Research Ethics Committee of the University of KwaZulu-Natal (study reference: BF078/08).

### 2.2 The APCDR EQ

The EQ is a C#-based program with supporting extensible markup language (XML) documentation containing the question list and visual formatting. Creation and alteration of question lists is done directly in XML, which is a programming language that allows the creation of design documents in a format that is both human and machine readable. As the main EQ program is coded separately from the question list, multiple XML documents can be created, allowing a single EQ program to run the same questionnaire with several language options or even several different questionnaires. Importantly, XML was specifically designed to emphasize simplicity and usability [5], providing a relatively low barrier to entry for the design and maintenance of questionnaires while still providing access to powerful options, such as Boolean logic question skips and automatic variable range control. Data collected by the EQ are stored in a MySQL database or Excel spreadsheet. Data transfer is facilitated through USB connections, avoiding the need for a constant Internet connection. Furthermore, the EQ program is being adapted for use across multiple platforms (eg, Android and Windows) and across multiple devices (eg, tablet, PC, and mobile phone). The source code for the EQ program and example XML documentation (Windows version) are freely available (https://github.com/apcdr/questionnaire) under the Affero General Public License free software license. Additionally, subsequent updated versions of the EQ, including additional applications such as audio recording and Global Positioning System capabilities, will also be available from the same Web site.

**Fig. 1:**
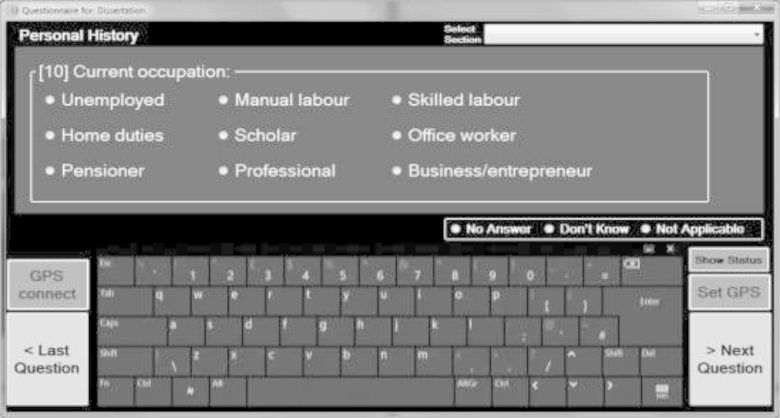
Sample electronic questionnaire screen presenting a multiple-choice question. GPS, Global Positioning System.

In this relative validation study, the question list consisted of 46 questions, which gathered information on sociodemographic indicators, medical history, and anthropometric measurements. We programed the EQ question list to duplicate the existing paper questionnaire from the T2DCC study as closely as possible (Appendix A; see at www.jclinepi.com). A sample screen of the EQ program is shown in Fig. 1.

### 2.3 Statistical analyses

We defined data collection errors as nonsensical or impossible inputs, missing data, or inputs that were inconsistent or incompatible with previous responses during the interview. Minor errors were classified a priori as differences of 1 year or less in date questions, whereas all remaining errors were classified as major errors. We calculated *P*-values for error comparisons between the EQ and paper methods using Pearson chi-square test or Fisher exact test, as appropriate. Cuzick's nonparametric test for trend across ordered groups was used to calculate *P*-values for the trend in duration of interviews over time. In all instances, *P*-values <0.05 (two sided) were considered statistically significant. Based on an error frequency estimate of one error per 100 questions for the traditional pen-and-paper method used in the T2DCC study, these analyses have more than 80% power to detect a change of 0.50 errors per 100 questions at the 5% level between the two methods. We based all salary cost analyses on formulae and estimates proposed by Walther et al. [6] for the comparison of electronic data capture systems with traditional pen-and-paper methods in an SSA country. The data were analyzed using Stata, version 11 (Stata Corporation, Texas, USA) and Microsoft Excel 2010.

## 3 Results

We collected data on 105 participants using the EQ and 95 participants using the paper method over 3 consecutive months. The slight discrepancy between groups was because of mistakes in implementing the randomization procedure; however, when we compared demographic characteristics between the EQ and paper randomization groups, we did not find any statistically significant differences (Appendix B; see at www.jclinepi.com).

The number of errors found for both the EQ and paper methods is presented in Table 1. The EQ had a lower number of major errors per 100 questions (EQ, 0.00 errors; paper, 0.59 errors; *P* < 0.001), as well as a lower overall number of errors per 100 questions (EQ, 0.17 errors; paper, 0.73 errors; *P* < 0.001). When using the pen-and-paper method, at least one error occurred every three interviews on average (33.4% of interviews contained at least one error), whereas for the EQ an error occurred once every 14 interviews on average (7.6% of interviews contained at least one error).

**Table 1:** Frequency of errors in 200 interviews, by month and method

Error type	Number of errors per 100 questions				Percent of interviews containing at least one error			
	Month 1	Month 2	Month 3	Overall	Month 1	Month 2	Month 3	Overall
All errors								
Paper	0.93	0.53	0.96	0.73	38.1	22.4	44.0	33.4
EQ	0.17	0.87	0.30	0.17∗	7.7	4.0	13.8	7.6
Major errors								
Paper	0.83	0.44	0.69	0.59	33.3	18.4	32.0	27.4
EQ	0.00	0.00	0.00	0.00∗	0.0	0.0	0.0	0.0
Minor errors								
Paper	0.10	0.89	0.26	0.14	4.8	4.1	12.0	6.3
EQ	0.17	0.87	0.30	0.17	7.7	4.0	13.8	7.6

*Abbreviation*: EQ, electronic questionnaire.

Minor errors classified as differences of 1 year or less in date calculations. Major errors classified as all other error types. *P*-values compare the number of errors for the EQ and paper methods for each error type using Pearson chi-square test.

∗ *P* ≤ 0.001.

Average duration of participant interviews, stratified by month and data collection method, is presented in Table 2. The overall average interview duration was similar for each method (EQ, 5.4 minutes; paper, 5.6 minutes), and the difference was not found to be statistically significant. There appeared to be no trend in interview duration over time for either data collection method.

**Table 2:** Duration of interview, by month and method (*n* = 200)

Method	Overall	Month 1	Month 2	Month 3	Trend over time
	Mean (range)				*P*-value
EQ (*n* = 105)	5.4 (3.7–8.8)	5.7 (4.2–8.8)	5.2 (4.0–7.4)	5.5 (3.7–8.7)	0.100
Paper (*n* = 95)	5.6 (2.0–15.0)	4.8 (2.0–7.0)	6.0 (4.0–15.0)	5.4 (4.0–10.0)	0.056

*Abbreviation*: EQ, electronic questionnaire.

Duration reported in minutes.

**Fig. 2:**
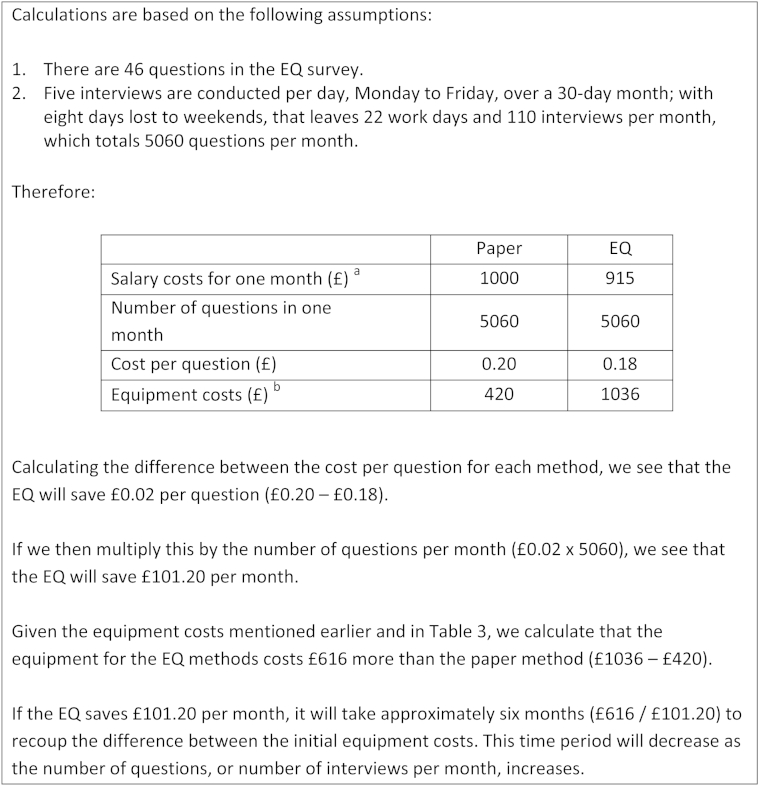
Worked example of estimated time taken to recoup costs using the EQ method. EQ, electronic questionnaire. ^a^Salary estimates are based on formulae presented by Walther et al. (see Table 3). ^b^Equipment costs are taken from Table 3.

A summary of estimated economic costs for each data collection method is presented in Table 3. All cost calculations were standardized to 1.00 for the paper method, for ease of comparison. Using the formulae proposed by Walther et al., the EQ salary cost per correctly entered question was almost half the cost of using pen-and-paper methods. In contrast, the initial technology costs for the EQ were 2.47 times larger than those estimated for paper methods. However, based on the costs incurred in the study presented here (Fig. 2), the EQ would recoup the difference in initial start-up costs within 6 months; this time frame would decrease if the number of questions, or the number of interviews per month, was increased.

**Table 3:** Estimated economic cost per method

Type of cost	Paper	EQ
Salary cost per correctly entered question^a^	1.00	0.51
Initial technology costs^b^	Desktop	Desktop + 2 tablets
	1.00	2.47
Additional overheads	Storage space for paper hard copies; office space for data entry clerk	Hardware maintenance and upkeep

*Abbreviation*: EQ, electronic questionnaire.

All costs standardized to 1.00 for the paper questionnaire.

a Salary costs per correctly entered question were calculated using the formulae presented by Walther et al. This includes the following assumptions: (1) Minimum staffing requirements are one field worker, one data entry clerk, and one data supervisor for the pen-and-paper method; and one field worker and one data manager for the EQ method. (2) Based on the EQ study budget, a data entry clerk would cost £250 per month. (3) Although no direct measurements were taken for the time required for double data entry and data quality control during the validation study, applicable estimates are available from published SSA studies. Based on these data, it was assumed that double data entry took 11.6 minutes and data quality control took 5 minutes per paper questionnaire, whereas data quality control took 3 minutes per EQ questionnaire.

b Technology costs are based on actual incurred costs of £308 per tablet and £420 per desktop.

## 4 Discussion

We describe an open-source adaptable electronic data capture system and compare it with traditional paper-based data collection methods in a cohort of 200 individuals. The results of this study suggest that the EQ may offer increased accuracy, similar interview duration, and increased data collection cost-effectiveness over paper-based methods. Overall, the APCDR EQ appears to offer a feasible and cost-effective alternative to paper-based data collection methods in SSA.

One advantage of the EQ is its increased data collection accuracy. Although both the EQ and paper methods contained a relatively low proportion of errors, the proportion of errors using the EQ system was less than a third of the proportion using the paper method. Additionally, all the EQ errors were classed as minor, whereas most of the errors made when using the paper method were considered major. Major errors, which included missing or nonsensical data, often require either dropping the piece of data in question or resurveying the participant to obtain corrected information, thus necessitating a choice between reduced statistical power or additional study costs. The increased accuracy of the EQ appears to reduce the frequency of such cost–benefit decisions.

Importantly, neither was there any statistically significant difference between the average interview durations for both methods nor was there a trend over time in interview duration for either data collection method. Previous research has proposed that, even assuming electronic data capture methods were as time efficient as paper methods, a lag would occur at the beginning of a study where using the electronic method would take markedly longer as study staff familiarized themselves with the system [6], [7]. There are several possible explanations why the data from the EQ validation study does not show this lag, including a well-run staff training program, software that was easy to use, or study staff who were already technically adept. Regardless, it does not appear that there is any appreciable time lag in data collection when moving from paper methods to the EQ in this setting.

Although determining the true economic cost efficiency of each data collection method is quite complex and study specific, this analysis has attempted to highlight and compare the major costs for both the EQ and paper methods. The EQ has higher start-up costs than the paper method, although it appears to have lower running costs once in place. Given that the initial start-up costs are a one-off expenditure, and the salary cost per correctly entered question is ongoing and cumulative, these data suggest that the EQ may be a cost-effective alternative to paper methods in longer running studies.

Our study has a number of strengths and potential limitations, which should be considered. We conducted this validation exercise within the context of an ongoing case–control study; our use of a pre-existing research questionnaire, real study participants, and the existing study team strengthens the relative validity of the study. We used a single staff member to administer the study questionnaires and so cannot exclude bias as a result of preference between the two data collection methods. Conversely, using a single staff member brought consistency across the duration of the study.

Our findings are in keeping with other studies that have shown the utility of electronic data capture and other informatics tools to facilitate data checks and early detection and correction of faulty procedures and data management [3]. For example, Singleton et al. [8], like us, identified that electronic capture systems often comprised web browsers, requiring keyboard and mouse input. These limitations led the authors to develop a touchscreen interface for their research studies, taking into consideration portability, limited screen space, and potentially inexperienced and low literacy users. This software, however, is not readily obtainable. By contrast—and with these same considerations in mind, which are particularly relevant to working in SSA and other low- and middle-income countries (LMICs)—we have developed our EQ as a publicly available resource and endeavored to overcome some of the barriers to using electronic data capture instruments identified by others [2], [9]. E-health technologies, and their evaluation, are becoming widespread in LMICs [10]; in this context, we anticipate that the benefits of easy-to-use simple electronic data capture systems such as the EQ will be increasingly realized.

Other electronic data capture systems are available in addition to the APCDR EQ. For example, programs such as REDCap and Microsoft Access, among others, have been used extensively for electronic data capture and storage. REDCap offers a secure web-based system of data capture, allowing the user to input data from anywhere in the world. It is widely used, with good technical support available and extensive training resources. Similarly, Microsoft Access is a commonly used program. Among its advantages, it is easy to integrate with other programs including non-Microsoft products such as Oracle and Sybase, and it has comprehensive technical support available on the Internet.

However, we created the APCDR EQ as our studies based in SSA highlighted certain limitations in existing data capture programs such as those mentioned previously. Specifically, programs such as REDCap require a constant Internet connection to be fully functioning, because of the online nature of the data collection program, and is not easy to customize. Microsoft Access requires licensing fees and does not give full access to the source code for expansion and adaptation of the software. By contrast, in developing the APCDR EQ, we aimed to provide a robust adaptable program that would operate at full capacity without an Internet connection, which was easy to maintain without compromising participant privacy and data security and thus was directly suited for use in challenging and isolated field environments such as in SSA.

In our study, the APCDR EQ appears to be more accurate and cost effective than currently used pen-and-paper methods, while maintaining data collection rates. With its multiplatform support and easily designed and maintained XML questionnaires, the EQ provides a simple, yet robust, option for data collection in resource-poor settings. We now plan to develop the EQ further, including exploring options for greater Internet connectivity and use of mobile networks to facilitate real-time data collection. The EQ software and supporting documentation are freely available (https://github.com/apcdr/questionnaire).

## Supplementary data

Supplementary Material

e-Appendix

## Footnotes

Supplementary data related to this article can be found at http://dx.doi.org/10.1016/j.jclinepi.2014.06.012.
