# Supplementary material for: Open-source electronic data capture system offered increased accuracy and cost-effectiveness compared with paper methods in Africa
Source: J Clin Epidemiol. 2014 Dec;67(12):1358–63. doi: 10.1016/j.jclinepi.2014.06.012 (PMC4271740; doi:10.1016/j.jclinepi.2014.06.012)
Supplement: Supplementary Material [file mmc1.pdf]

Appendix A. T2DCC Paper Questionnaire

Collection of samples from subjects of Zulu descent with and without type 2 diabetes for studies of the genetic basis of diabetes in African populations.

DATA FORM: TYPE 2 DIABETES Stick label here

DATE OF STUDY (DD / MM / YYYY) ☐☐ ☐☐ ☐☐☐☐

Hospital / Clinic name: ____

Hospital number: ☐☐☐☐☐☐☐☐ Study number: ☐☐☐☐☐

Has informed consent been signed? Yes = 1; No = 2 ☐

Do not proceed with the study if informed consent has not been signed

Surname: ☐

Initials: ☐☐☐ Title: ☐

Address: ☐

☐

Telephone: ☐

Remove this front page from the remainder to de-identify

Appendix A. T2DCC Paper Questionnaire

Collection of samples from subjects of Zulu descent with and without type 2 diabetes for studies of the genetic basis of diabetes in African populations.

DATA FORM: TYPE 2 DIABETES

DATE OF STUDY (DD / MM / YYYY) ☐☐ ☐☐ ☐☐☐☐

START TIME OF THE QUESTIONNAIRE (HH:MM) ☐☐ ☐☐

Study number: ☐☐☐☐☐

Stick label here

Has informed consent been signed? Yes = 1; No = 2 ☐

Do not proceed with the study if informed consent has not been signed

PERSONAL HISTORY:

Date of birth: ☐☐ ☐☐ ☐☐☐☐ Age (years) ☐ Gender ☐

DD MM YYYY

Male = 1

Female = 2

Highest educational level ☐

Primary school =1

High school = 2

Tertiary institute = 3

Appendix A. T2DCC Paper Questionnaire

Alcohol consumption (units per week): (1-100)

(1 unit of alcohol is defined as 330 mL beer, 200 mL wine or 50 mL spirits)

Cigarette smoking (pack years): (1-100)

(1 pack year is defined as 10 cigarettes daily for 1 year)

Current occupation:

Unemployed =1; Manual labour = 2; Skilled labour = 3; Home duties = 4;

Scholar = 5; Office worker = 6; Pensioner = 7;

Professional = 8; Business / entrepreneur = 9;

Occupational physical activity

Sedentary = 1; light = 2; moderate = 3; heavy = 4

Leisure physical activity

Never = 1; Less than once weekly = 2; 1-2 times weekly = 3; > 3 times weekly = 4

VERIFICATION OF ZULU ORIGIN:

First language Zulu

Yes = 1; No = 2

Both parents Zulu

Yes = 1; No = 2

FAMILY HISTORY:

Diabetes in parents:

Yes = 1; No = 2; Do not know = 3

If yes, specify

Appendix A. T2DCC Paper Questionnaire

Father = 1; Mother = 2; Both parents = 3

Diabetes in grandparents

Yes = 1; No = 2; Do not know = 3

If yes, specify

Paternal grandfather = 1; Paternal grandmother = 2

Maternal grandfather = 3; Maternal grandmother = 4

Maternal and paternal grandparent affected = 5

Number of siblings:

Diabetes in siblings:

Yes = 1; No = 2; Do not know = 3

If yes, specify

Brother = 1; Sister = 2

If brother(s) affected, specify number:

If sister(s) affected, specify number:

DIABETES MELLITUS:

Year of diagnosis

Age at diagnosis (years)

Current therapy

None = 1; oral therapy = 2; insulin alone = 3; Oral plus insulin = 4

Appendix A. T2DCC Paper Questionnaire

If on insulin, date of commencement of insulin therapy (year)

Lipid-lowering therapy

Yes = 1; No = 2; Do not know = 3

CLINICAL INFORMATION:

Co-morbid illnesses:

None = 1; Respiratory = 2; Cardiovascular = 3

Gastrointestinal = 4; Renal = 5; Neurologic = 6; Other = 7

Nature of co-morbid illness

Infectious = 1; Non-infectious = 2

Macrovascular disease

Coronary artery disease: Yes = 1; No = 2

Cerebrovascular disease: Yes = 1; No = 2

Peripheral vascular disease: Yes = 1; No = 2

Hypertension: Yes = 1; No = 2

If yes, year of diagnosis

Microvascular disease

Retinopathy: Yes = 1; No = 2

Appendix A. T2DCC Paper Questionnaire

Nephropathy: Yes = 1; No = 2

Neuropathy: Yes = 1; No = 2

CLINICAL MEASUREMENTS

Height (cm)

Weight (kg)

Body mass index (kg/m^2)

Waist circumference (cm)

Hip circumference (cm)

Waist-Hip ratio

Systolic blood pressure - 1 (mmHg)

Systolic blood pressure – 2 (mmHg)

Diastolic blood pressure - 1 (mmHg)

Diastolic blood pressure – 2 (mmHg)

Appendix A. T2DCC Paper Questionnaire

END TIME OF THE QUESTIONNAIRE (HH:MM) ☐☐ ☐☐

Appendix B. Summary of Demographic Characteristics, by Interview Method

	EQ	Paper	T-test for difference
	n (%)	n (%)	*P* value
Number of individuals	105	95	
Sex			0.122
Men	22 (21%)	29 (30.5%)	
Women	83 (79%)	66 (69.5%)	
Education			0.587
None	3 (2.9%)	6 (6.3%)	
Primary school	51 (48.6%)	33 (34.7%)	
High school	46 (43.8%)	49 (51.6%)	
Tertiary institute	5 (4.8%)	7 (7.4%)	
	mean (SD)	mean (SD)	p-value
Age (years)	55.5 (8.8)	55.7 (9.3)	0.361
Alcohol (units per week)	0.29 (1.4)	0.08 (0.50)	0.189
Smoking (pack years)	0.14 (0.54)	0.03 (0.18)	0.059

SD=Standard deviation. Pack year is defined as 10 cigarettes daily for one year. Smoking status and alcohol consumption from self-reported information
