# Supplementary material for: Open-source electronic data capture system offered increased accuracy and cost-effectiveness compared with paper methods in Africa
Source: J Clin Epidemiol. 2014 Dec;67(12):1358–63. doi: 10.1016/j.jclinepi.2014.06.012 (PMC4271740; doi:10.1016/j.jclinepi.2014.06.012)
Supplement: e-Appendix [file mmc2.docx]

**Response to reviewers’ comments**

**JCE-13-751**

E-appendix: Acknowledgements

EHY had full access to all the data collected for the study, and had final responsibility for the decision to submit for publication. DGD, MSS, and EHY conceived the study concept and design. DGD and CP oversaw the creation of the EQ programme. DGD, AAM and FP oversaw study implementation and data collection. All authors took part in the interpretation of the data. DGD, MSS, and EHY drafted the Article, and all authors provided critical revisions of the Article for important intellectual content. We would like to further acknowledge Stephen Rice, who wrote the majority of the APCDR EQ programme, Nonhlanhla Moloantola for conducting the participant interviews, and to all the staff and study participants of the T2DCC study in Durban, for contributing the data used in this study. Membership of the African Partnership for Chronic Disease Research: Clement Adebamowo, Adewbowale Adeyemo, Morris Agaba, Albert Amoah, Felix Assah, Naby Balde, Inês Barroso, Joram Buza, Bilkish Cassim, Tobias Chirwa, Francis Collins, Nigel Crowther, Frank Dudbridge, Tonya Esterhuizen, Heiner Grosskurth, Andrew Haines, Sophie Hawkesworth, Branwen J. Hennig, Robert Heyderman, Shabbar Jaffar, Pontiano Kaleebu, Anatoli Kamali, Saidi Kapiga, Elly Katabira, Kerstin Klipstein-Grobusch, Dominic Kwiatkowski, Naomi S. Levitt, Edna Majaliwa, Patricia Marshall, Fredrick Mashili, Mary Mayige, Jean Claude Mbanya, Mark McCarthy, Sophie E. Moore, Andrew Morris, Ayesha Motala, Paula Munderi, Marina Njelekela, Shane Norris, Ousman Nyan, Moffat Nyirenda, John Oli, Michael Parker, Nasheeta Peer, Fraser Pirie, Andrew M. Prentice, Kaushik Ramaiya, Raj Ramasar, Michele Ramsay, Charles Rotimi, Manjinder Sandhu, Janet Seeley, Liam Smeeth, Eugene Sobngwi, Steve Tollman, Nicholas Wareham, Elizabeth Young, Eleftheria Zeggini
